# The Secretome of Bone Marrow and Wharton Jelly Derived Mesenchymal Stem Cells Induces Differentiation and Neurite Outgrowth in SH-SY5Y Cells

**DOI:** 10.1155/2014/438352

**Published:** 2014-07-15

**Authors:** Ana O. Pires, Andreia Neves-Carvalho, Nuno Sousa, António J. Salgado

**Affiliations:** ^1^Life and Health Sciences Research Institute (ICVS), School of Health Sciences, University of Minho, Campus de Gualtar, 4710-057 Braga, Portugal; ^2^ICVS/3B's, PT Government Associate Laboratory, Braga/Guimarães, Portugal

## Abstract

The goal of this study was to determine and compare the effects of the secretome of mesenchymal stem cells (MSCs) isolated from human bone-marrow (BMSCs) and the Wharton jelly surrounding the vein and arteries of the umbilical cord (human umbilical cord perivascular cells (HUCPVCs)) on the survival and differentiation of a human neuroblastoma cell line (SH-SY5Y). For this purpose, SH-SY5Y cells were differentiated with conditioned media (CM) from the MSCs populations referred above. Retinoic acid cultured cells were used as control for neuronal differentiated SH-SY5Y cells. SH-SY5Y cells viability assessment revealed that the secretome of BMSCs and HUCPVCs, in the form of CM, was able to induce their survival. Moreover, immunocytochemical experiments showed that CM from both MSCs was capable of inducing neuronal differentiation of SH-SY5Y cells. Finally, neurite lengths assessment and quantitative real-time reverse-transcription polymerase chain reaction (RT-PCR) analysis demonstrated that CM from BMSCs and HUCPVCs differently induced neurite outgrowth and mRNA levels of neuronal markers exhibited by SH-SY5Y cells. Overall, our results show that the secretome of both BMSCs and HUCPVCs was capable of supporting SH-SY5Y cells survival and promoting their differentiation towards a neuronal phenotype.

## 1. Introduction

Central nervous system (CNS) neurological disorders/injuries often pose a major challenge for treatment due to the limited capability of CNS to self-renew and to regenerate [1]. These CNS features have prompted the search for new therapies, such as those using mesenchymal stem cells (MSCs). MSCs have been defined as multipotent cells which are capable of self-renewal [2]. Additionally, they are known to adhere to tissue culture flasks and to display the presence of MSCs surface markers (CD105, CD73, and CD90), as well as the lack of hematopoietic MSCs cell surface markers (CD45, CD34, CD14 or CD11b, CD79a or CD19 and human leukocyte antigen DR) [2, 3]. Current sources of MSCs include bone marrow, adipose tissue, dental pulp, placenta, amniotic fluid, umbilical cord blood, umbilical cord Wharton's jelly, liver, lung, and spleen [3, 4].

MSCs isolated from different sources have been proposed for CNS related applications. Indeed, MSCs transplantation has shown to have a therapeutic effect in animal models of ischemia [5, 6], spinal cord injury (SCI) [7, 8], and Parkinson's disease (PD) [9, 10]. The underlying mechanisms by which the MSCs transplantation mediates the beneficial outcomes remain to be elucidated. Although the putative MSCs differentiation into neuronal lineages has been purposed as the major contributor for CNS regeneration in animal models of neurodegenerative diseases [11–15], MSCs differentiation into full functional neuronal lineages remains to be clarified [16–18]. In contrast, robust data indicates that CNS tissue restorative effects are mediated by MSCs secretome, that is, the panel of bioactive factors and vesicles, with neuroregulatory properties, released by these cells to the extracellular environment [10, 19–42].

For instance, we have demonstrated that human BMSCs secretome promotes cell survival and increases cell viability of rat postnatal hippocampal neurons and cortical glial cells [19]. Nakano et al. also showed that the secretome of BMSCs cultured in the supernatant of ischemic brain extracts was able to increase neuronal survival and neurite outgrowth of postnatal rat hippocampal neurons, through apoptosis suppression mechanisms [20]. These findings were correlated with the expression and secretion of IGF-1 (insulin-like growth factor 1), HGF (hepatocyte growth factor), VEGF (vascular endothelial growth factor), and TGF *β*1 (transforming growth factor beta 1) by the BMSCs [20]. This was further confirmed by other studies in which, upon being cultured with extracts from ischemic and traumatic brain, BMSCs altered its gene expression profile when compared with uninjured control brain extracts [21, 22]. Moreover, significant improvements in functional recovery were also described in* in vivo* models of ischemia, upon intravenous injection of BMSCs [23–25]. In these studies, improvements in neurologic function were accompanied by a reduction of infarct size and/or with an increase in endogenous cell proliferation and a reduction of apoptosis. These neuroprotective and neurorecovery effects have thus been attributed to BMSCs secretion of interleukin-6 (IL-6) neurotrophic and anti-inflammatory cytokine as well as of growth factors (GFs) such as nerve growth factor (NGF), brain-derived neurotrophic factor (BDNF), glial cell-derived neurotrophic factor (GDNF), VEGF, TGF *β*1, IGF-1, insulin-like growth factor 2 (IGF 2), epidermal growth factor (EGF), and basic fibroblast growth factor (bFGF).

Similar findings were also reported in* in vitro* [26–29] and* in vivo* [30–32] models of spinal cord injury. For instance, Führman et al. [28] and Gu et al. [29] reported that coculture of BMSCs with dorsal root ganglia (DRG) explants and neurons significantly enhanced neuronal cell survival and neurite outgrowth, through the secretion of NGF, BDNF, bFGF, and CNTF (ciliary neurotrophic factor), HGF, SDF-1 (stromal cell-derived factor 1), VEGF, EGF, NT-3 (neurotrophin-3), and NT-4 (neurotrophin 4) GFs, as well as IL-1 (interleukin-1), IL-6, and IL-8 (interleukin-8) cytokines. This expression pattern is in accordance with data published by others upon BMSCs transplantation in animal models of SCI [30–32]. On the other hand, several authors have also reported that BMSCs expression of BDNF, GDNF, EGF, bFGF, VEGF, HGF, SDF-1, and NT-3 could be correlated with dopaminergic (DAergic) neurons protection against 6-hydroxydopamine (6-OHDA) neurotoxin both in* in vitro* and* in vivo* models of PD [33–35].

Similarly, the secretome of MSCs isolated from the Wharton's jelly of the umbilical cord (WJ-MSCs) also disclosed some interesting properties for CNS regenerative medicine. For instance, Ribeiro et al. [36] and Fraga et al. [37] revealed that the secretome of mesenchymal progenitors isolated from the Wharton's jelly of the umbilical cord increased neuronal cell viability and cell densities. These effects were attributed to the expression of NGF and the vesicular fraction of the secretome, respectively, which contained proteins typically involved in neuroprotection. Several studies also revealed that the expression of neuroprotective, neurogenic, and angiogenic GFs as well as of growth-associated cytokines, like BDNF, GDNF, bFGF, G-CSF (granulocyte colony-stimulating factor), SDF-1, PDGF-AA (platelet-derived growth factor AA), angiopoietin-2, VEGF receptor 3 (VEGF-R3), CXCL-16 (chemokine ligand 16), and NAP-2 (neutrophil-activating protein-2), could be correlated with WJ-MSCs beneficial outcomes towards ischemic stroke in rats [38–40]. On the other hand, Yang et al. [41] and Hu et al. [42] linked the improvement of locomotor function, the neuroprotection, and the axon regeneration in a rat SCI model with the WJ-MSCs secretion of NT-3, GDNF, bFGF, VEGF-R3, NAP-52 (neutrophil-activating protein-52), and GITR (glucocorticoid-induced tumor necrosis factor receptor). Finally, Weiss et al. [10] showed that WJ-MSCs transplantation could also ameliorate the condition of a hemi-Parkinsonian rat model through the secretion of GDNF and FGF 20 (fibroblast growth factor 20) DAergic trophic factors.

Despite all these studies, there are few reports where the effects of the secretome of MSCs, isolated from different sources, on neuronal cell populations are directly compared. Therefore, in the present study we aimed to determine to which extent the secretome of MSCs isolated from the bone marrow and the connective tissue surrounding umbilical cord vessels affected the survival and differentiation of a human neuroblastoma cell line. Our results show that the secretome of BMSCs and HUCPVCs, in the form of CM, is able* per se* to induce SH-SY5Y cells survival, differentiation into neuron-like cells, and neurite outgrowth. Moreover, the secretome of BMSCs and HUCPVCs, collected at different time points, was capable of increasing SH-SY5Y neuronal differentiation at the same extent as the retinoic acid (RA), which is commonly used to differentiate SH-SY5Y cells [43]. Finally, CM from BMSCs and HUCPVCs displayed different temporal profiles regarding stimulation of neurite outgrowth and the gene expression of neuronal markers exhibited by SH-SY5Y cells.

## 2. Materials and Methods

### 2.1. Cell Culture

#### 2.1.1. Bone Marrow Tissue Derived Stem Cells

BMSCs were acquired from PROMOCELL (Heidelberg, Germany). Cells were thawed and expanded according to the protocol previously described by Silva et al. [44]. Briefly, BMSCs were cultured in *α*-MEM (GIBCO, Grand Island, NY, USA) supplemented with sodium bicarbonate (NaHCO_3_, MERCK, USA), 10% of fetal bovine serum (FBS, BIOCHROM AG, UK), and 1% of penicillin-streptomycin antibiotic (GIBCO). Confluent cells were trypsinised, plated in new T75 tissue culture flasks (NUNC, Denmark), at a density of 4.000 cells/cm^2^, and incubated at 37°C in a 5% humidified CO_2_ atmosphere. The culture medium was changed every two to three days. BMSCs were used for experiments during passage 6 (P6).

#### 2.1.2. Human Umbilical Cord Perivascular Cells

HUCPVCs were kindly provided by Professor J. E. Davies (University of Toronto, Canada). Cells isolation from umbilical cord was performed according to the procedures described by Sarugaser and coworkers [45]. Expansion of cells was performed according to the protocol described above for BMSCs. HUCPVCs were used for experiments during P6.

#### 2.1.3. Human Neuroblastoma Cell Line

SH-SY5Y cells were cultured following the methods previously published by Lopes et al. [46]. Briefly, cells were thawed and grown in T75 flasks (NUNC) containing Dulbecco's modified Eagles medium nutrient mixture F12 (DMEM/F-12, PAA, LABCLINICS, M, Spain), to which were added 1% of glutamax (GIBCO), 10% of FBS (BIOCHROM AG), and 1% of kanamycin sulfate (GIBCO). Confluent cells were trypsinized and plated at a density of 42.105 cells/cm^2^ in 13 mm glass coverslips, double precoated with both poly-D-lysine (SIGMA-ALDRICH, St. Louis, MO, USA) and pig skin gelatin (SIGMA-ALDRICH), inserted in 24-well plates (NUNC) for cell metabolic viability, immunocytochemical, and neurite outgrowth assays. For analysis of SH-SY5Y cells gene expression regarding several neuronal markers, cells were plated in 6-well plates (NUNC) at a density of 42.105 cells/cm^2^. Afterwards, cells were incubated with the same medium described above in a 5% humidified CO_2_ atmosphere at 37°C for 24 h, after which media were changed and the experiments were performed as described below. SH-SY5Y cells were used for experiments between passages 11 and 15.

#### 2.1.4. Conditioned Media Collection and Experiments

CM was collected from P6 BMSCs and HUCPVCs as previously reported by Fraga et al. [37]. Shortly, cells were plated at a density of 4.000 cells/cm^2^ and allowed to grow for 3 days in a 5% humidified CO_2_ atmosphere at 37°C. Culture medium was then renewed and collected 24 h and 96 h thereafter (cell culture was not renewed or added during this time period). Collected CM were frozen and thawed only in the day of experiments. For CM collection, DMEM/F-12 media supplemented with 1% of glutamax and 1% of kanamycin sulfate were used.

For differentiation assays, SH-SY5Y cells were incubated with BMSCs or HUCPVCs CM and respective positive control for neuronal differentiation (SH-SY5Y cells were cultured with DMEM/F-12 (PAA, LABCLINICS) supplemented with 1% of glutamax (GIBCO), 1% of FBS (BIOCHROM AG), 1% of kanamycin sulfate (GIBCO), and 10 *μ*M of RA (SIGMA-ALDRICH)). An additional group, SH-SY5Y proliferative/undifferentiated cells, was also carried out (see Supplementary Data in Supplementary Material available online at http://dx.doi.org/10.1155/2014/438352). The culture medium was changed every day for 7 days, in the end of which cell metabolic viability, differentiation, and neurite outgrowth were assessed in parallel with all other experimental conditions.

### 2.2. Cell Viability Assessment

Cell metabolic viability was assessed by the MTS test. The MTS [3-(4,5-dimethylthiazol-2-yl)-5-(3-carboxymethoxyphenyl)-2-(4-sulfophenyl)-2H-tetrazolium] test (PROMEGA, Madison, WI, USA) is a cell viability assay based on the bioreduction of the substrate (MTS) to a brown formazan product. Cell culture coverslips (*n* = 3 replicates) were set in culture medium containing MTS in a 5 : 1 ratio and incubated at 37°C with 5% humidified CO_2_ atmosphere. Three hours after incubation, 100 *μ*L of each sample was transferred to 96-well plates (*n* = 3 replicates) and optical density (OD) was measured at a 490 nm.

### 2.3. Immunocytochemistry

*Cells were cultured in double precoated coverslips (n* = 5), fixed with 4% paraformaldehyde (MERCK, USA), and incubated for 30 min at room temperature (RT). After incubation, cells were permeabilised by incubation with 0.3% triton X-100 (MERCK)/PBS 1x (GIBCO). Membrane receptors were then blocked for 60 min (RT) with 10% FBS (BIOCHROM AG)/PBS. Afterwards, cells were incubated (60 min) with mouse anti-rat microtubule-associated protein 2 (MAP-2) antibody (SIGMA-ALDRICH) to detect mature SH-SY5Y neurons. Cells were washed thereafter with 0.5% FBS/PBS solution and incubated for 60 min (RT) with Alexa Fluor 488 goat anti-mouse immunoglobulin G. Finally, samples were incubated for 5 min with DAPI (4′,6-diamidino-2-phenylindole dihydrochloride), to stain cells nuclei (THERMO SCIENTIFIC, Rockford, USA), and observed under an OLYMPUS IX-81 fluorescence microscope (OLYMPUS, Germany).

### 2.4. Cell Counts

For cell counts, five representative fields of each coverslip condition (*n* = 5 replicates) were selected with 20x magnification and analyzed using Cell-P software (OLYMPUS, Germany). In addition, according to the literature, MAP-2 positive cells with one or more neurites were counted as differentiated cells [47].

### 2.5. Neurite Lengths Assessment

For neurite lengths assessment in SH-SY5Y neuron-like cells, multiple representative fields of cells morphology stained with MAP-2 labeling were photographed with an IX-81 OLYMPUS fluorescence microscope (OLYMPUS, Germany) fitted to a DP-711 digital camera (OLYMPUS, Germany). Captured images were labeled with a scale according to the correspondent microscope magnification (40x). The images scale was used to convert pixels units into micrometers (*μ*m), using for this purpose the NIH Image J (Rasband WS, Image J, NIH), version 1.41. In addition, the channels were extracted to grey scale and the length of 5 to 10 neurites per field was traced and measured, thereafter, from the distal end of neuron growth-cone, using the neurite tracer plugin of NIH Image [48, 49].

### 2.6. Quantitative Real-Time RT-PCR

Total cellular ribonucleic acid (RNA) was extracted from SH-SY5Y differentiated cells with RA or MSCs CM (*n* = 3 replicates), using Trizol reagent (APPLIED BIOSYSTEMS, Life Technologies, CA, USA) for cell lysis and chloroform (MERCK)/isopropanol (THERMO SCIENTIFIC) for RNA isolation. The amount of RNA extracted and its purity were determined by measuring OD at 260 nm and 280 nm in ND-1000 spectrophotometer (ALFAGENE, PT). RNA was then treated with ribonuclease (RNAse) free desoxirribonuclease (DNAse, THERMO SCIENTIFIC) and 1 *μ*g of total RNA was reverse-transcribed using Superscript kit (BIO-RAD, CA, USA) to obtain complementary deoxyribonucleic acid (cDNA). After obtaining cDNA, 1 *μ*g of cDNA per reaction was amplified by quantitative real-time PCR in a CFX96 detection system (BIO-RAD) by means of SSOfast Evagreen supermix (BIO-RAD) and the primers sequences (concentration of 1 *μ*M) previously described, using an annealing temperature of 60°C [50]. Each aliquot of cDNA was subjected to 40 PCR amplification cycles (94°C for 20 s, primer annealing at 60°C for 30 s, extension at 72°C for 40 s). Primers sequences used corresponded to several genes, namely, synaptophysin, *β*III tubulin, MAP-2, DRD2 (dopamine receptor D2), and DAT (dopamine transporter). The expression levels of neuronal markers were determined as previously reported [51].

### 2.7. Statistical Analysis

Statistical evaluation was performed using one-way ANOVA followed by* Bonferroni's* post hoc test to assess statistical correlation between retinoic acid-differentiated cells (RA-differentiated cells) and conditioned media groups (for statistical evaluation, 3 replicates of each sample were used to perform the MTS test and RT-PCR, whereas five replicates were used to assess immunocytochemical and neurite outgrowth data (*n* = 3/*n* = 5; RA-differentiated cells/CM time point ± SEM)). These statistical tests were complemented with student's *t-*test to determine statistical correlation between RA and conditioned media groups (*n* = 3/*n* = 5; RA-differentiated cells/CM time point ± SEM) or between conditioned media groups corresponding to the same time point (*n* = 3/*n* = 5; CM time point ± SEM). Statistical significance was defined as *P* < 0.05 for a 95% confidence interval.

## 3. Results and Discussion

**Figure 1:**
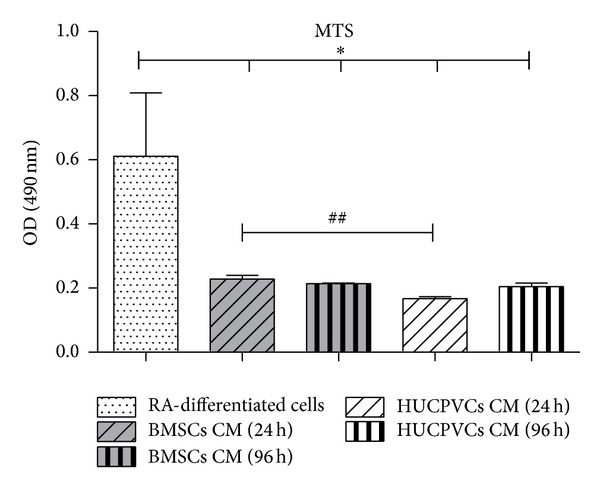
Metabolic viability (MTS test) of SH-SY5Y cells seven days after incubation with BMSCs and HUCPVCs CM. Results revealed that, for all CM tested conditions, the secretome of both MSCs populations is able to support neuronal-like cell viability without the use of any other exogenous growth factors. The differences observed towards RA-differentiated cells (*P* < 0.05) are considered to be natural as these cultures were supplemented with 1% of FBS. Concerning statistical differences among time points, BMSCs CM 24 h promoted a significant increase in SH-SY5Y cells viability when compared with the HUCPVCs CM 24 h group (*P* < 0.01) (values are shown as mean ± SEM, *n* = 3). Symbols correspondence to statistical signification: (1) ∗ refers to comparisons between RA-differentiated cells and MSCs CM; (2) # regards the correlation between MSCs CM from the same time point (^##^ *P* < 0.01, *P < 0.05).

In the present study we aimed to determine and compare how the secretome of two MSCs populations, derived from either the bone marrow or the Wharton Jelly surrounding umbilical cord vessels, could impact the viability and neuronal differentiation of a human neuroblastoma cell line. For this purpose, SH-SY5Y cells incubated with a combination of low percentage of FBS and RA treatment were used as positive control of SH-SY5Y cells differentiation (RA-differentiated cells). Results revealed that cells incubated with both BMSCs and HUCPVCs CM had similar levels of metabolic viability after 7 days of culture (Figure 1). However, the values were significantly lower (*P* < 0.05) than those obtained for control samples. These differences were within the expected as control cultures were incubated with 1% of FBS, which can increase their metabolic viability. Of note is the fact that SH-SY5Y cells were not able to survive for more than 5 days of* in vitro* culture when incubated in plain neurobasal media, without the addition of any other supplements (CM control; data not shown). This fact is a strong indicator that,* per se*, the secretome of both MSCs populations is able to support neuronal-like cell viability, without the use of any other exogenous growth factors.

**Figure 2:**
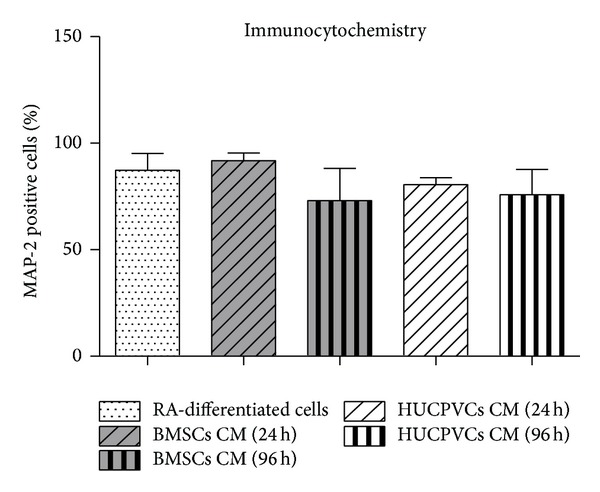
Cell densities for MAP-2 positive cells presenting neurites seven days after incubation with BMSCs and HUCPVCs CM. Immunocytochemistry assessment (using Cell-P software and 20x magnification micrographs) revealed that all CM tested conditions presented similar percentages of MAP-2 positive cells when compared to RA-differentiated cells, the positive control for SH-SY5Y differentiation (*P* > 0.05). The later effect was more noticeable for the BMSCs CM 24 h. Therefore, these results show that the secretome of both BMSCs and HUCPVCs is capable of inducing SH-SY5Y cells differentiation into neuronal-like cells (values are shown as mean ± SEM, *n* = 5, and statistical significance was defined as *P* < 0.05).

**Figure 3:**
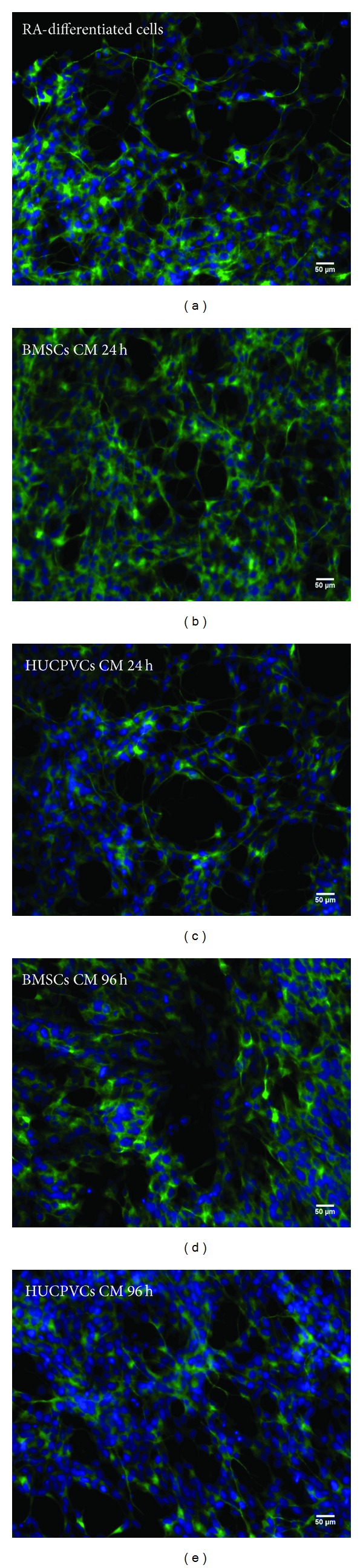
Fluorescence microscopy micrographs of SH-SY5Y cells immunostained with MAP-2 seven days after incubation with RA (a), BMSCs CM (24 h, 96 h: (b), (d)), and HUCPVCs CM (24 h, 96 h: (c), (e)). As it can be observed, the secretome of both BMSCs and HUCPVCs was able to induce SH-SY5Y cells differentiation into neuronal-like cells.

Following this initial cell viability assay, an analysis on the differentiation of SH-SY5Y cells incubated with BMSCs/HUCPVCs CM or RA was performed by determining the percentage of cells positive for the neuronal marker MAP-2 that displayed one or more neurites (Figure 2). This criterion was established according to what was previously described by Encinas et al. [47]. Results revealed that all CM incubated groups had similar percentages of differentiated SH-SY5Y cells when compared to the positive control for cell differentiation (RA-differentiated cells, *P* > 0.05) (Figure 2 and Figures 3(a) to 3(e)). This effect was more noticeable for the BMSCs CM 24 h (Figure 2 and Figure 3(b)). Thus, from the data obtained, it is possible to state that the secretome of both BMSCs and HUCPVCs is capable of inducing SH-SY5Y cells neuronal differentiation.

**Figure 4:**
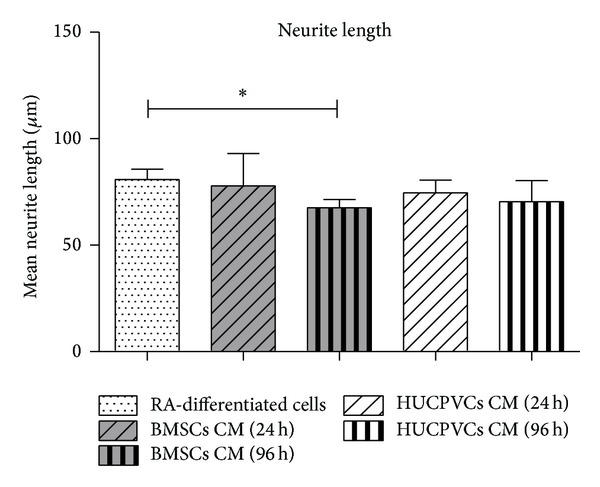
Quantitative analysis of SH-SY5Y neurite outgrowth seven days after incubation with BMSCs and HUCPVCs CM. Quantification of neurite lengths showed that for all CM conditions, with exception of SH-SY5Y cells cultured with BMSCs CM 96 h, the CM of BMSCs and HUCPVCs induced a neurite outgrowth and length very similar to the RA-differentiated cells group (*P* > 0.05). The decrease in the mean neurite length observed in the BMSCs CM 96 h group (*P* < 0.05) suggests that the neuronal differentiation inducement of SH-SY5Y cells may be associated with the temporal profile of MSCs CM collection (values are shown as mean ± SEM, *n* = 5, *P < 0.05).

**Figure 5:**
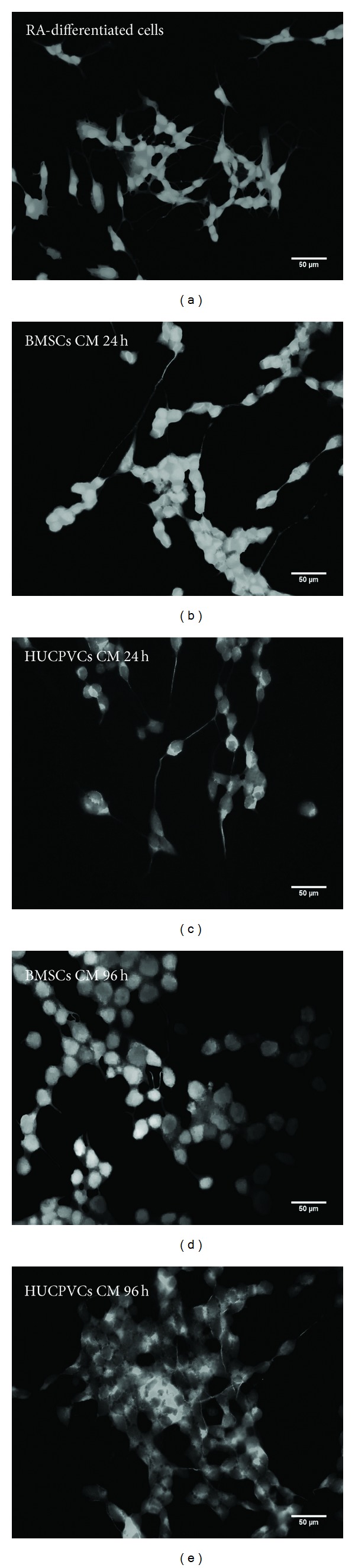
Representative micrographs (magnification: 40x) used to quantify neurite outgrowth in SH-SY5Y cells seven days after incubation with RA (a), BMSCs CM (24 h, 96 h: (b), (d)), and HUCPVCs CM (24 h, 96 h: (c), (e)) through the use of neurite tracer plugin from Image J.

In order to further understand the role of the CM of HUCPVCs and BMSCs on SH-SY5Y neuronal differentiation, a quantitative analysis of neurite lengths was carried out (Figure 4). As it can be observed, BMSCs CM 24 h as well as HUCPVCs CM 24 h and 96 h had very similar results to those of the RA-differentiated cells group (*P* > 0.05) (Figure 4 and Figures 5(a) to 5(c) and 5(e)), which is a strong indicator of the differentiation effects of the secretome of both BMSCs and HUCPVCs. Finally, the decrease in the mean neurite length observed in the BMSCs CM 96 h group (*P* < 0.05) (Figure 4 and Figures 5(a) and 5(d)) may be related with the half-life of neurotrophic factors present in the CM at the point of their collection [19, 36, 37].

**Figure 6:**
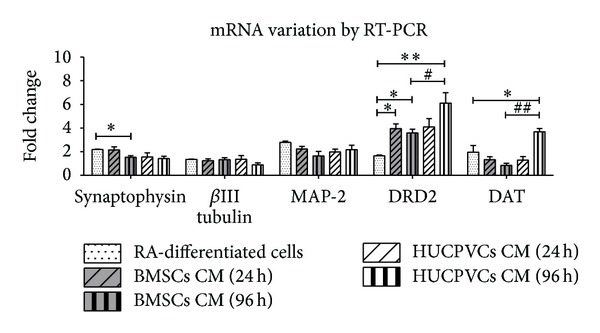
Variation of neuronal markers seven days after incubation with BMSCs and HUCPVCs CM. Levels of mRNA for different neuronal markers were quantified by quantitative real-time RT-PCR and normalized to both undifferentiated/proliferative cells (reference level: 1) and HBMS housekeeping gene. Quantification of neuronal markers expression revealed that BMSCs CM 24 h and 96 h displayed a significant increase in the SH-SY5Y cells expression of DRD2 gene when compared to RA-differentiated cells (*P* < 0.05). However, BMSCs CM 96 h simultaneously induced a decrease in synaptophysin in comparison with RA-differentiated cells (*P* < 0.05). On the other hand, for all the neuronal markers studied, no statistically significant differences were found between HUCPVCs CM 24 h and RA groups (*P* > 0.05). Yet, HUCPVCs CM 96 h significantly elevated mRNA levels of DRD2 and DAT genes when compared with both BMSCs 96 h (*P* < 0.05; *P* < 0.01) and RA-differentiated cells (*P* < 0.01; *P* < 0.05). The later results suggest that HUCPVCs CM 96 h is inducing SH-SY5Y cells towards the DAergic phenotype. In addition, differences between BMSCs and HUCPVCs from the same time point indicate that different tissue derived MSCs secretome have distinct effects in SH-SY5Y cells differentiation with respect to neuronal phenotype. For all the other neuronal markers studied, no significant statistical differences were observed between all CM tested conditions and RA-differentiated cells (*P* > 0.05), which strongly indicates that the CM from BMSCs and HUCPVCs are capable of inducing SH-SY5Y cells neuronal differentiation. Moreover, the different expression pattern of neuronal markers exhibited by SH-SY5Y cells among CM time points of collection indicates that the effects mediated by MSCs secretome in SH-SY5Y cells differentiation is related with the temporal profile of CM collection (values are shown as mean ± SEM, *n* = 3). Symbols correspondence to statistical signification: (1) ∗ refers to comparisons between RA-differentiated cells and MSCs CM and (2) # regards the correlation between MSCs CM from the same time point (^∗∗/##^ *P* < 0.01, ^∗/#^ *P* < 0.05).

To confirm SH-SY5Y cells differentiation towards neuronal phenotype, seven days after incubation with CM from BMSCs and HUCVCs, mRNA expression of several neuronal specific markers was assessed by quantitative RT-PCR. According to the literature, mRNA levels of dopamine transporter and receptor D2 as well as levels of vesicle proteins (e.g., synaptophysin), neuronal specific cytoskeletal proteins (e.g., MAP-2), and globular proteins (e.g., *β*III tubulin) were found increased in SH-SY5Y cells upon differentiation with RA [50, 52]. As it can be observed in Figure 6, the mRNA level of DRD2 was significantly increased in SH-SY5Y cells differentiated with BMSCs CM 24 h when compared to RA-differentiated cells (*P* < 0.05). Similarly, DRD2 gene expression was significantly elevated in SH-SY5Y cells differentiated with BMSCs CM 96 h when compared to RA-differentiated cells (*P* < 0.05). On the other hand, for all the neuronal markers studied, no statistically significant differences for SH-SY5Y cells gene expression were found between HUCPVCs CM 24 h and RA groups (*P* > 0.05). Interestingly, SH-SY5Y cells differentiation with HUCPVCs CM 96 h resulted in a significant increase in DRD2 and DAT genes expression in comparison with RA-differentiated cells (*P* < 0.01, *P* < 0.05). As DAT is a gene expressed only by DAergic neurons [53], this result suggests that GFs present in HUCPVCs CM 96 h may be inducing SH-SY5Y cells towards the DAergic phenotype. Indeed, it has been reported that SH-SY5Y cells differentiate into the cholinergic, adrenergic, or DAergic phenotype depending on media conditions [52]. In addition, the different SH-SY5Y cells expression pattern observed between HUCPVCs CM 24 h and HUCPVCs 96 h further reinforces the hypothesis that different temporal profiles of CM collection have distinct effects on SH-SY5Y cells differentiation. For all the other neuronal markers studied, no significant differences were found among the different tested CM conditions and RA groups (*P* > 0.05). These results further reinforce that the secretome of both BMSCs and HUCPVCs induces neuronal differentiation of SH-SY5Y cells. RT-PCR results also revealed differences regarding mRNA levels of DRD2 and DAT genes between BMSCs and HUCPVCs, collected at the same time point (96 h). Indeed, SH-SY5Y cells differentiated with HUCPVCs CM 96 h exhibited significant greater expression of both DRD2 and DAT genes than cells differentiated with BMSCs CM 96 h (*P* < 0.05, *P* < 0.01). The later result suggests not only that the different secretome composition of different tissue derived MSCs induces SH-SY5Y cells differentiation into different neuronal phenotypes but also that the effects mediated by the secretome of MSCs in neuronal differentiation are associated with the temporal profile of CM collection [19, 36, 37].

## 4. Conclusions

The present study has shown that the secretome of both BMSCs and HUCPVCs was capable of supporting SH-SY5Y cell survival, while promoting their differentiation towards a neuronal phenotype. Furthermore, it was also observed that the secretome collected from both MSCs populations may induce SH-SY5Y cells differentiation into different neuronal phenotypes, which is an indicator of possible differences within the secretome of the two cell populations. Therefore, future studies should not only provide full characterization of factors secreted by MSCs derived from different microenvironments/sources but also assess the impact that different temporal profile of secretome collection can hold towards different CNS pathologies/injuries. Additionally the effects of cell passaging on MSCs secretome should also be assessed.

## Supplementary Material

The supplementary materials contain supplementary methods (cell metabolic viability, immunocytochemical, quantitative RT-PCR and neurite outgrowth assays), statistics, figures (representing the results obtained) and discussion regarding the use of low percentage of fetal bovine serum and retinoic acid as positive control of SH-SY5Y cells differentiation. Overall, results show that retinoic acid-differentiated cells not only provide a good positive control for proliferative cells differentiation, but are also a suitable control for SH-SY5Y cells differentiated with CM from both BMSCs and HUCPVCs.

## References

[1] Emsley JG, Mitchell BD, Kempermann G, Macklis JD (2005). Adult neurogenesis and repair of the adult CNS with neural progenitors, precursors, and stem cells. *Progress in Neurobiology*.

[2] Dominici M, Le Blanc K, Mueller I (2006). Minimal criteria for defining multipotent mesenchymal stromal cells. The International Society for Cellular Therapy position statement. *Cytotherapy*.

[3] Teixeira FG, Carvalho MM, Sousa N, Salgado AJ (2013). Mesenchymal stem cells secretome: a new paradigm for central nervous system regeneration?. *Cellular and Molecular Life Sciences*.

[4] Carvalho MM, Teixeira FG, Reis RL, Sousa N, Salgado AJ (2011). Mesenchymal stem cells in the umbilical cord: phenotypic characterization, secretome and applications in central nervous system regenerative medicine. *Current Stem Cell Research and Therapy*.

[5] Ohtaki H, Ylostalo JH, Foraker JE (2008). Stem/progenitor cells from bone marrow decrease neuronal death in global ischemia by modulation of inflammatory/immune responses. *Proceedings of the National Academy of Sciences of the United States of America*.

[6] Hirko AC, Dallasen R, Jomura S, Xu Y (2008). Modulation of inflammatory responses after global ischemia by transplanted umbilical cord matrix stem cells. *Stem Cells*.

[7] Cizkova D, Rosocha J, Vanicky I, Jergova S, Cizek M (2006). Transplants of human mesenchymal stem cells improve functional recovery after spinal cord injury in the rat. *Cellular and Molecular Neurobiology*.

[8] Kyung HL, Suh-Kim H, Ji SC (2007). Human mesenchymal stem cell transplantation promotes functional recovery following acute spinal cord injury in rats. *Acta Neurobiologiae Experimentalis*.

[9] Bouchez G, Sensebé L, Vourc’h P (2008). Partial recovery of dopaminergic pathway after graft of adult mesenchymal stem cells in a rat model of Parkinson’s disease. *Neurochemistry International*.

[10] Weiss ML, Medicetty S, Bledsoe AR (2006). Human umbilical cord matrix stem cells: preliminary characterization and effect of transplantation in a rodent model of Parkinson’s disease. *Stem Cells*.

[11] Offen D, Barhum Y, Levy Y-S (2007). Intrastriatal transplantation of mouse bone marrow-derived stem cells improves motor behavior in a mouse model of Parkinson’s disease. *Journal of Neural Transmission, Supplementa*.

[12] Bahat-Stroomza M, Barhum Y, Levy YS (2009). Induction of adult human bone marrow mesenchymal stromal cells into functional astrocyte-like cells: potential for restorative treatment in parkinson’s disease. *Journal of Molecular Neuroscience*.

[13] Shetty P, Ravindran G, Sarang S, Thakur AM, Rao HS, Viswanathan C (2009). Clinical grade mesenchymal stem cells transdifferentiated under xenofree conditions alleviates motor deficiencies in a rat model of Parkinson’s disease. *Cell Biology International*.

[14] Hayase M, Kitada M, Wakao S (2009). Committed neural progenitor cells derived from genetically modified bone marrow stromal cells ameliorate deficits in a rat model of stroke. *Journal of Cerebral Blood Flow and Metabolism*.

[15] Pedram MS, Dehghan MM, Soleimani M, Sharifi D, Marjanmehr SH, Nasiri Z (2010). Transplantation of a combination of autologous neural differentiated and undifferentiated mesenchymal stem cells into injured spinal cord of rats. *Spinal Cord*.

[16] Kolf CM, Cho E, Tuan RS (2007). Mesenchymal stromal cells. Biology of adult mesenchymal stem cells: regulation of niche, self-renewal and differentiation. *Arthritis Research and Therapy*.

[17] Maltman DJ, Hardy SA, Przyborski SA (2011). Role of mesenchymal stem cells in neurogenesis and nervous system repair. *Neurochemistry International*.

[18] Liu J, Song L, Jiang C (2012). Electrophysiological properties and synaptic function of mesenchymal stem cells during neurogenic differentiation—a mini-review. *International Journal of Artificial Organs*.

[19] Ribeiro CA, Salgado AJ, Fraga JS, Silva NA, Reis RL, Sousa N (2011). The secretome of bone marrow mesenchymal stem cells-conditioned media varies with time and drives a distinct effect on mature neurons and glial cells (primary cultures). *Journal of Tissue Engineering and Regenerative Medicine*.

[20] Nakano N, Nakai Y, Seo T-B (2010). Characterization of conditioned medium of cultured bone marrow stromal cells. *Neuroscience Letters*.

[21] Chen X, Katakowski M, Li Y (2002). Human bone marrow stromal cell cultures conditioned by traumatic brain tissue extracts: growth factor production. *Journal of Neuroscience Research*.

[22] Qu R, Li Y, Gao Q (2007). Neurotrophic and growth factor gene expression profiling of mouse bone marrow stromal cells induced by ischemic brain extracts. *Neuropathology*.

[23] Li Y, Chen J, Chen XG (2002). Human marrow stromal cell therapy for stroke in rat: neurotrophins and functional recovery. *Neurology*.

[24] Mackay KB, Loddick SA, Naeve GS, Vana AM, Verge GM, Foster AC (2003). Neuroprotective effects of insulin-like growth factor-binding protein ligand inhibitors in vitro and in vivo. *Journal of Cerebral Blood Flow and Metabolism*.

[25] Wakabayashi K, Nagai A, Sheikh AM (2010). Transplantation of human mesenchymal stem cells promotes functional improvement and increased expression of neurotrophic factors in a rat focal cerebral ischemia model. *Journal of Neuroscience Research*.

[26] Wright KT, El Masri W, Osman A (2007). Bone marrow stromal cells stimulate neurite outgrowth over neural proteoglycans (CSPG), myelin associated glycoprotein and Nogo-A. *Biochemical and Biophysical Research Communications*.

[27] Kamei N, Tanaka N, Oishi Y (2007). Bone marrow stromal cells promoting corticospinal axon growth through the release of humoral factors in organotypic cocultures in neonatal rats. *Journal of Neurosurgery: Spine*.

[28] Führmann T, Montzka K, Hillen LM (2010). Axon growth-promoting properties of human bone marrow mesenchymal stromal cells. *Neuroscience Letters*.

[29] Gu Y, Wang J, Ding F, Hu N, Wang Y, Gu X (2010). Neurotrophic actions of bone marrow stromal cells on primary culture of dorsal root ganglion tissues and neurons. *Journal of Molecular Neuroscience*.

[30] Lu P, Jones LL, Tuszynski MH (2005). BDNF-expressing marrow stromal cells support extensive axonal growth at sites of spinal cord injury. *Experimental Neurology*.

[31] Neuhuber B, Timothy Himes B, Shumsky JS, Gallo G, Fischer I (2005). Axon growth and recovery of function supported by human bone marrow stromal cells in the injured spinal cord exhibit donor variations. *Brain Research*.

[32] Himes BT, Neuhuber B, Coleman C (2006). Recovery of function following grafting of human bone marrow-derived stromal cells into the injured spinal cord. *Neurorehabilitation and Neural Repair*.

[33] Shintani A, Nakao N, Kakishita K, Itakura T (2007). Protection of dopamine neurons by bone marrow stromal cells. *Brain Research*.

[34] Wang F, Yasuhara T, Shingo T (2010). Intravenous administration of mesenchymal stem cells exerts therapeutic effects on parkinsonian model of rats: focusing on neuroprotective effects of stromal cell-derived factor-1*α*. *BMC Neuroscience*.

[35] Cova L, Armentero M-T, Zennaro E (2010). Multiple neurogenic and neurorescue effects of human mesenchymal stem cell after transplantation in an experimental model of Parkinson’s disease. *Brain Research*.

[36] Ribeiro CA, Fraga JS, Grãos M (2012). The secretome of stem cells isolated from the adipose tissue and Wharton jelly acts differently on central nervous system derived cell populations. *Stem Cell Research and Therapy*.

[37] Fraga JS, Silva NA, Lourenço AS (2013). Unveiling the effects of the secretome of mesenchymal progenitors from the umbilical cord in different neuronal cell populations. *Biochimie*.

[38] Ding D-C, Shyu W-C, Chiang M-F (2007). Enhancement of neuroplasticity through upregulation of *β*1-integrin in human umbilical cord-derived stromal cell implanted stroke model. *Neurobiology of Disease*.

[39] Koh S-H, Kim KS, Choi MR (2008). Implantation of human umbilical cord-derived mesenchymal stem cells as a neuroprotective therapy for ischemic stroke in rats. *Brain Research*.

[40] Lin Y-C, Ko T-L, Shih Y-H (2011). Human umbilical mesenchymal stem cells promote recovery after ischemic stroke. *Stroke*.

[41] Yang C-C, Shih Y-H, Ko M-H, Hsu S-Y, Cheng H, Fu Y-S (2008). Transplantation of human umbilical mesenchymal stem cells from Wharton’s jelly after complete transection of the rat spinal cord. *PLoS ONE*.

[42] Hu S-L, Luo H-S, Li J-T (2010). Functional recovery in acute traumatic spinal cord injury after transplantation of human umbilical cord mesenchymal stem cells. *Critical Care Medicine*.

[43] Blomhoff R, Blomhoff HK (2006). Overview of retinoid metabolism and function. *Journal of Neurobiology*.

[44] Silva NA, Gimble JM, Sousa N, Reis RL, Salgado AJ (2013). Combining adult stem cells and olfactory ensheathing cells: the secretome effect. *Stem Cells and Development*.

[45] Sarugaser R, Lickorish D, Baksh D, Hosseini MM, Davies JE (2005). Human umbilical cord perivascular (HUCPV) cells: a source of mesenchymal progenitors. *Stem Cells*.

[46] Lopes FM, Schröder R, da Frota ML (2010). Comparison between proliferative and neuron-like SH-SY5Y cells as an in vitro model for Parkinson disease studies. *Brain Research*.

[47] Encinas M, Iglesias M, Liu Y (2000). Sequential treatment of SH-SY5Y cells with retinoic acid and brain-derived neurotrophic factor gives rise to fully differentiated, neurotrophic factor-dependent, human neuron-like cells. *Journal of Neurochemistry*.

[48] Wenker SD, Chamorro ME, Vota DM, Callero MA, Vittori DC, Nesse AB (2010). Differential antiapoptotic effect of erythropoietin on undifferentiated and retinoic acid-differentiated SH-SY5Y cells. *Journal of Cellular Biochemistry*.

[49] Pool M, Thiemann J, Bar-Or A, Fournier AE (2008). NeuriteTracer: a novel ImageJ plugin for automated quantification of neurite outgrowth. *Journal of Neuroscience Methods*.

[50] Constantinescu R, Constantinescu AT, Reichmann H, Janetzky B (2007). Neuronal differentiation and long-term culture of the human neuroblastoma line SH-SY5Y. *Journal of Neural Transmission, Supplementa*.

[51] Livak KJ, Schmittgen TD (2001). Analysis of relative gene expression data using real-time quantitative PCR and the 2-ΔΔCT method. *Methods*.

[52] Xie H-R, Hu L-S, Li G-Y (2010). SH-SY5Y human neuroblastoma cell line: in vitro cell model of dopaminergic neurons in Parkinson’s disease. *Chinese Medical Journal*.

[53] Storch A, Hwang Y-I, Gearhart DA (2004). Dopamine transporter-mediated cytotoxicity of *β*-carbolinium derivatives related to Parkinson’s disease: relationship to transporter-dependent uptake. *Journal of Neurochemistry*.

