# Supplementary material for: The Secretome of Bone Marrow and Wharton Jelly Derived Mesenchymal Stem Cells Induces Differentiation and Neurite Outgrowth in SH-SY5Y Cells
Source: Stem Cells Int. 2014 Jul 15;2014:438352. doi: 10.1155/2014/438352 (PMC4124228; doi:10.1155/2014/438352)
Supplement: Supplementary file 1 — The supplementary materials contain supplementary methods (cell metabolic viability, immunocytochemical, quantitative RT-PCR and neurite outgrowth assays), statistics, figures (representing the results obtained) and discussion regarding the use of low percentage of fetal bovine serum and retinoic acid as positive control of SH-SY5Y cells differentiation. Overall, results show that retinoic acid-differentiated cells not only provide a good positive control for proliferative cells differentiation, but are also a suitable control for SH-SY5Y cells differentiated with CM from both BMSCs and HUCPVCs. [file 438352.f1.pdf]

Supplementary data

Supplementary methods

Proliferative cells culture and experiments

A control of SH-SY5Y proliferative/undifferentiated cells was also performed during the seven days differentiation of SH-SY5Y cells with MSCs CM. For this purpose, SH-SY5Y cells were cultured in DMEM/F-12 (PAA, LABCLINICS®, M, Spain) supplemented with 10 % of FBS (BIOCHROM AG®), 1% of glutamax (GIBCO®, Grand Island, NY, USA) and 1% of kanamycin sulfate (GIBCO®) for seven days. The culture medium was changed every day during this period of time. In addition, seven days later, cell metabolic viability (MTS test), immunocytochemical, RT-PCR and neurite outgrowth assays were performed at the same time as all the other tested conditions, following the same procedures described in the section of Materials and Methods. In addition, SH-SY5Y proliferative/undifferentiated cells were compared with RA-differentiated cells regarding cells metabolic viability, differentiation and neurite outgrowth, in order to demonstrate that RA-differentiated cells provide a good positive control for the objectives of this work.

Supplementary notes

Statistics

Statistical evaluation was performed using *t-test* to assess statistical differences between proliferative cells and RA-differentiated cells (for statistical evaluation 3 replicates of each sample were used to perform the MTS test, whereas five replicates were used to assess

immunocytochemical and neurite lengths data; n = 3 / n = 5; proliferative cells /RA-differentiated cells ±SD).

Supplementary figures

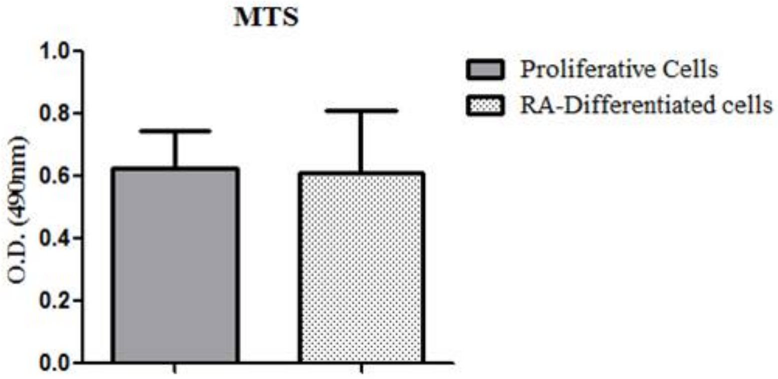

Suppl. Fig. 1: Metabolic Viability of SH-SY5Y cells seven days post-incubation with FBS and RA. Results revealed that RA-differentiated cells were able to support SH-SY5Y cells viability at the same extension as SH-SY5Y proliferative cells (Values are shown as mean ± SD, n = 3, statistical significance was defined as $p<0.05$).

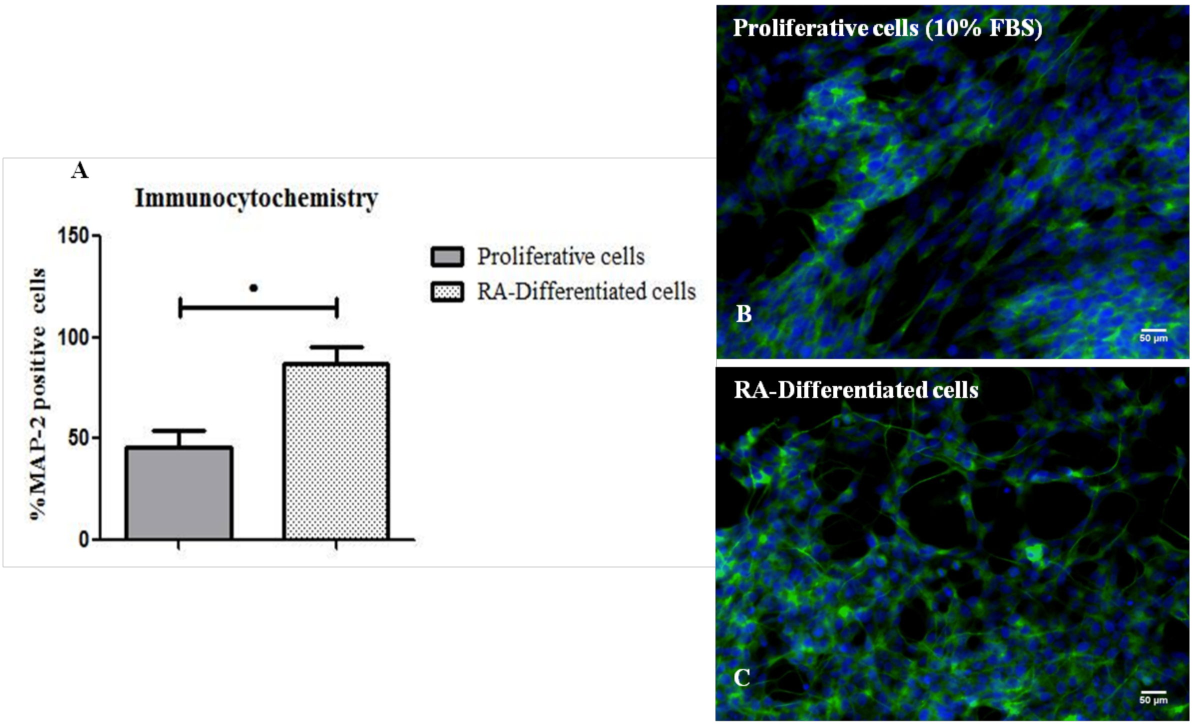

Suppl. Fig. 2: Cell densities for MAP-2 positive cells presenting neurites seven days post-incubation with FBS and RA. Cell densities assessment revealed that the densities of MAP-2 positive cells after culture with RA (A, C) where significantly higher than the ones presented by SH-SY5Y proliferative cells (A, B) (Values are shown as mean ± SD, n = 5, • $p<0.05$).

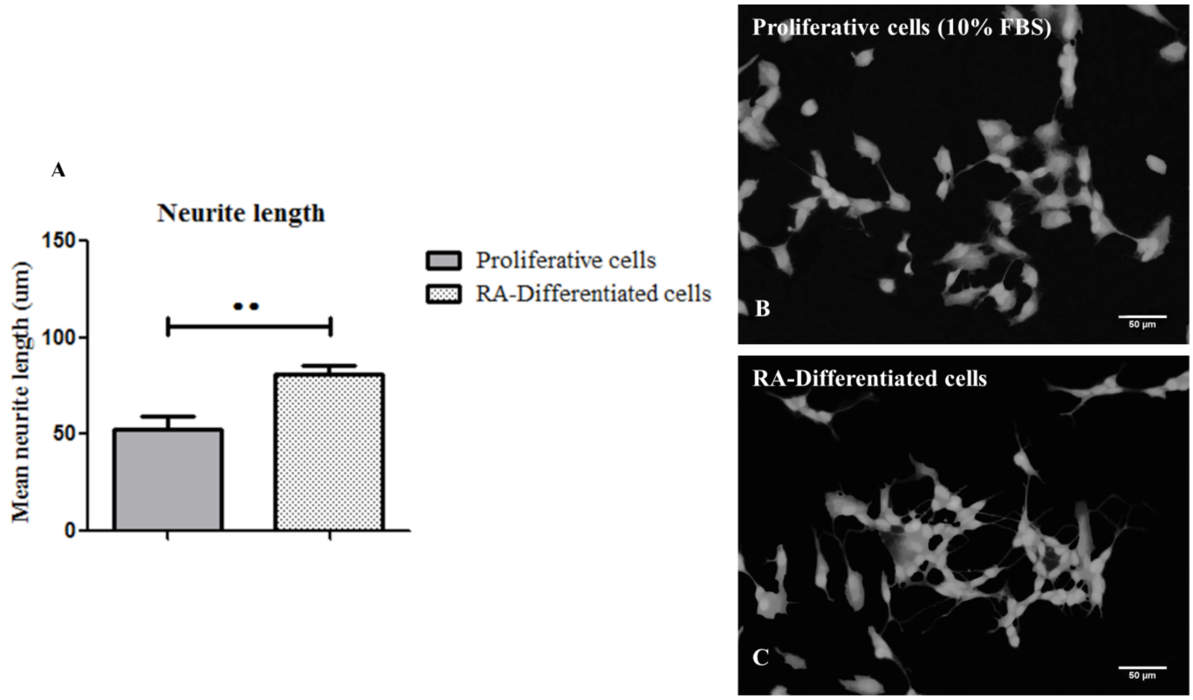

Suppl. Fig. 3: Quantitative analysis of SH-SY5Y neurite outgrowth seven days post-incubation with FBS and RA. Neurite lengths assessment revealed that RA-differentiated cells (A, C) stimulated neurite outgrowth at a greater extent than SH-SY5Y proliferative cells (A, B) (Values are shown as mean ± SD, n = 5, ** p<0.01).

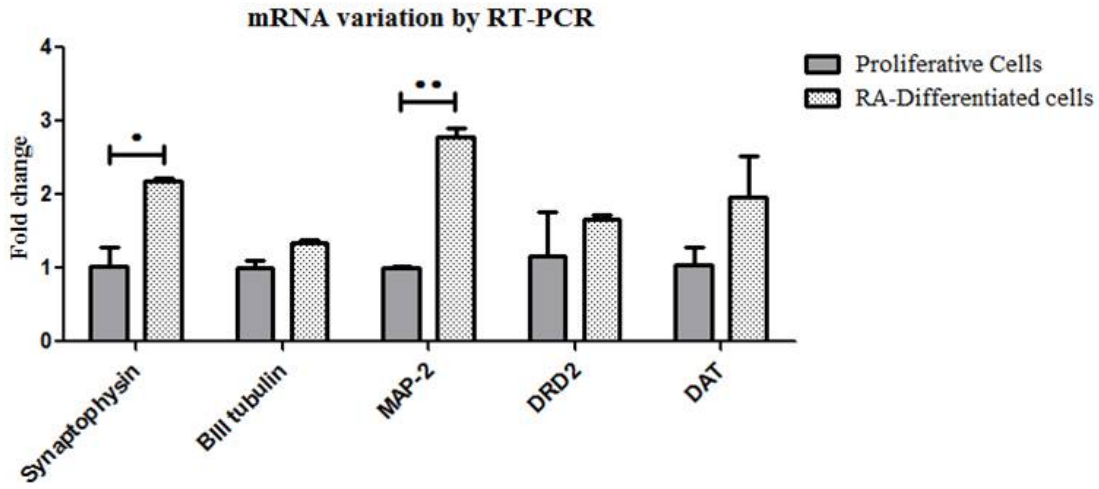

Suppl. Fig. 4: Variation of neuronal markers seven days post-incubation with FBS and RA. Levels of mRNA for different neuronal markers was quantified by quantitative real-time RT-PCR, normalized to undifferentiated/proliferative cells (reference level: 1) and HBMS housekeeping gene. Quantification of neuronal markers expression revealed that the mRNA levels of Synaptophysin and MAP-2 were significantly increased in RA-differentiated cells when compared to the expression levels of SH-SY5Y proliferative cells ($p<0.05$, $p<0.01$). For all the other neuronal markers, an increase in expression was also noticed, although not at statistically significant level (Values are shown as mean ± SD, n = 3, • $p<0.05$, •• $p<0.01$).

Supplementary discussion

It has been largely documented that a combination of low percentage of FBS and RA induces SH-SY5Y cells differentiation into neuron-like cells [29-31, 33]. Therefore, in the present study, a control of SH-SY5Y proliferative/undifferentiated cells was provided to confirm that SH-SY5Y cells were differentiated seven days post-incubation with 1% FBS and RA. Results revealed that RA-differentiated cells presented similar cell viability as SH-SY5Y proliferative cells (Figure S 1; $p>0.05$). Also, immunocytochemical and neurite lengths data revealed that SH-SY5Y cells differentiated with RA exhibited significant higher densities of MAP-2 positive cells (Figure S 2; $p<0.05$,) and mean neurite lengths (Figure S 3; $p<0.01$). Moreover, Synaptophysin and MAP-2 gene expression levels were significantly increased in RA-differentiated cells in comparison with SH-SY5Y proliferative cells (Figure S 4; $p<0.05$, $p<0.01$). For all the other neuronal markers, an increase SH-SY5Y cells genes expression was also noticed although not at a statistically significant level.

Taken together, results show that the differentiation protocol, using low percentage of FBS and RA, effectively induced SH-SY5Y proliferative cells differentiation into neuron-like

cells. Therefore RA-differentiated cells provide a good positive control for the objectives of this work.
